# Illness trajectory in the longer term after hospitalisation for COVID-19: a prospective, multicentre cohort study

**DOI:** 10.1186/s12879-025-12487-w

**Published:** 2026-01-20

**Authors:** Anna Kamdar, Andrew J. Morrow, Robert Sykes, Alasdair McIntosh, Catherine Bagot, Hannah K. Bayes, Kevin G. Blyth, Colin Church, Lynsey Gillespie, Giles Roditi, David Stobo, Sarah Weeden, Paul Welsh, Kenneth Mangion, Alex McConnachie, Colin Berry

**Affiliations:** 1 School of Cardiovascular and Metabolic Health, University of Glasgow, 126 University Place, Glasgow, G12 8TA UK; 2 Department of Cardiology, Queen Elizabeth University Hospital, Greater Glasgow and Clyde Health Board, Glasgow, G51 4TF UK; 3 Robertson Centre for Biostatistics, School of Health and Wellbeing, University of Glasgow, Glasgow, G11 6EW UK; 4 Department of Haematology, Glasgow Royal Infirmary, Greater Glasgow and Clyde Health Board, G4 0SF Glasgow, UK; 5 Department of Respiratory Medicine, Glasgow Royal Infirmary, Greater Glasgow and Clyde Health Board, Glasgow, G4 0SF UK; 6 Department of Respiratory Medicine, Queen Elizabeth University Hospital, Greater Glasgow and Clyde Health Board, Glasgow, G51 4TF UK; 7 Project Management Unit, Greater Glasgow and Clyde Health Board, Glasgow, G51 4TF UK; 8 Department of Radiology, Glasgow Royal Infirmary, Greater Glasgow and Clyde Health Board Glasgow, Glasgow, G4 0SF UK; 9 Department of Radiology, Queen Elizabeth University Hospital, Greater Glasgow and Clyde Health Board, Glasgow, G51 4TF UK

**Keywords:** Viral infection, COVID-19, Comorbidities, Cardiovascular, Respiratory

## Abstract

**Background:**

There are few data on the longer-term illness trajectory of patients following hospitalisation for COVID-19.

**Methods:**

We prospectively enrolled 267 adults hospitalised for COVID-19. Longer-term follow up was available for 260 participants. Event rates for death or unplanned hospitalisation were calculated using a Poisson model. Univariate and multivariable analyses identified baseline predictors, with a backward selection process for the best fitting model.

**Results:**

The mean age of COVID-19 participants was 54.9±12.1 years, and 41% were female. During median follow-up of 1028 days (IQR:1000,1085), 112 individuals (43.1%) had at least one event including 6 deaths (2.3%). There were 252 events in total. The first event rate was 18.9 per 100 person-years (95%CI: 15.7, 22.8). Multivariable predictors included healthcare worker status (HR 0.59, 95%CI: 0.34, 1.02, p=0.046), Charlson Comorbidity Index (HR 1.13, 95%CI: 1.02, 1.24, p=0.020), current smoking (HR 2.49, 95%CI: 1.21, 5.11, p=0.010), and haemoglobin (HR 0.93, 95%CI: 0.88, 0.99, p=0.020). The WHO Clinical Severity Score was not a significant predictor (p=0.187).

**Conclusion:**

Comorbidity, current smoking status and haemoglobin predict illness trajectory following hospitalisation for COVID-19, rather than illness severity during hospitalisation. Further research is needed to explore interventions targeting these factors to improve prognosis.

**Trial registration:**

CISCO-19; http://NCT04403607. Registration date; 23/05/2020

**Supplementary Information:**

The online version contains supplementary material available at 10.1186/s12879-025-12487-w.

## Background

The COVID-19 pandemic has had a profound and enduring impact on global public health, leading to widespread illness, significant mortality, and overwhelming healthcare systems. While much of the focus has been on the acute management of COVID-19, particularly during the early stages of the pandemic, less attention has been given to prognosis in the longer term, i.e. > 1-year, for rehospitalisation and death. Now, five years on, many survivors continue to face ongoing health difficulties, with repeated hospital admissions and an elevated risk of mortality [1–3].

Longer-term follow-up of patients infected with the Omicron COVID-19 variant in China demonstrates that measurable symptoms and functional limitations can endure for years [4]. At the population level, increased healthcare utilisation among people with long COVID reflects sustained pressures placed on individuals and health systems alike [5]. This emerging area of work highlights that the long-term consequences of COVID-19 remain clinically significant, demographically varied, and systemically burdensome. However, much of the research conducted so far has centred on relatively short-term outcomes in registry or database populations [6–8] or excluded those with a readmission [9].

We investigated the longer-term illness trajectory of patients after hospitalisation with COVID-19, focusing on all-cause mortality and readmission to hospital. We aimed to identify the factors that predict events since this information may be helpful to inform healthcare strategies and guide resource allocation for longer term follow-up care.

## Methods

The CISCO-19 study (http://NCT04403607) was a prospective, multicentre cohort study involving post-COVID-19 patients enrolled either during the index hospitalisation or at the time of discharge in three hospitals in the West of Scotland (population 2.2 million). The study methods [10] and primary outcome [11] have been previously reported.

The inclusion criteria for the CISCO-19 study were; (1) age ≥18 years; (2) history of an unplanned hospital visit (for example, emergency department or hospitalisation > 24 hours) for COVID-19 confirmed by a laboratory test for COVID-19 participants; (3) ability to comply with study procedures; and (4) ability to provide written informed consent. The exclusion criteria were: (1) contraindication to MRI (for example, severe claustrophobia or metallic foreign body) and (2) lack of informed consent. The first study visit (enrolment) involved informed consent and assessments during the initial hospitalisation or as soon as possible after discharge.

### Charlson comorbidity index

The Charlson Comorbidity Index was calculated for each participant [12]. Higher scores indicate greater comorbidity and were used as a baseline predictor of death or unplanned hospitalisation.

### ISARIC 4c score

The ISARIC Comprehensive Clinical Characterization Collaboration (4c) [13, 14] quantifies risk of mortality in hospitalized COVID-19 patients based on age, comorbidities, physiological parameters, and laboratory values. The percentage score was calculated at baseline and used as a predictor of death or unplanned hospitalisation.

### COVID-19 participants

Patients who received hospital care for COVID-19, with or without admission, and were alive, were prospectively screened in real time using an electronic healthcare information system (TrakCare, InterSystems) and daily hospital reports identifying inpatients with laboratory-positive results for COVID-19. A diagnosis of COVID-19 was based on laboratory evidence of SARS-CoV-2 infection using a PCR test (Roche Cobas 6800 or Seegene SARS-CoV-2 PCR) [11, 12].

### Controls

The rationale for a control group was to compare baseline characteristics of the post-COVID-19 population to assess for selection bias. The control group eligibility criteria included a history of cardiovascular risk factors and/or hospitalisation for non COVID-19 respiratory illness. The contemporary control group was prospectively recruited by public advertising during the study period.

Control participants with similar age, sex, ethnicity and cardiovascular risk factors underwent the same research procedures during a single visit between 13 April and 2 July 2021.

Inclusion criteria for controls were; age 40–80 years and at least one cardiovascular risk factor from the ASSIGN criteria [15]. Exclusion criteria were a prior history of myocardial infarction, myocarditis, heart failure, structural heart disease, positive serology for COVID-19 or a history of COVID-19.

Medical research staff screened the electronic health records of patients under their care, or personal contacts, with known episodes of care in primary or secondary care. Before the research visit, a blood test for COVID-19 serology (Abbott Architect CMIA SARS-CoV-2 IgG assay) was used to confirm the absence of prior infection with COVID-19. All of the controls had negative serology tests for COVID-19. A blood test for COVID-19 serology. Controls were not included in the prognostic analysis.

### Clinical outcomes

The early clinical outcomes [11] and the study protocol [10] have been previously reported. In brief, the study involved the prospective collection of clinical data and a time-course of research investigations. Clinical data including demographics, medical and cardiovascular history were prospectively recorded at enrolment.

Clinical research team members assessed study participants’ electronic health records and prospectively recorded events. Clinical outcomes included death and unplanned episodes of care. The causes of unplanned hospitalisations and death were determined based on adjudication by clinicians who were independent of the attending clinical team. Death was defined as death from any cause occurring during follow-up, regardless of prior hospitalisations. Unplanned respiratory hospitalisations were adjudicated by the clinical events committee and classified as non-COVID-19 respiratory hospitalisations when the primary reason for admission was a respiratory condition in the absence of confirmed COVID-19 infection. An unplanned hospitalisation was defined as an episode of unscheduled care (e.g. emergency department, acute medical receiving ward), which led to overnight/next day stay or longer.

### Statistical analysis

Person-years of follow-up were calculated from the date of index COVID-19 hospital discharge to the earliest of the following events; date of first unplanned episode of care, date of death, or date of clinical record review. One person-year is equivalent to one person being followed for one year. First-event rates per 100 patient-years were calculated using a Poisson model while confidence intervals for total event rates per 100 patient-years were calculated using negative binomial regression.

Univariate analyses were conducted to assess the association between each predictor and the risk of unplanned hospitalisation or death. Predictors with a p-value < 0.10 in univariate analysis were considered for inclusion in a multivariable analysis. The proportional hazards assumption was checked by examination of Schoenfeld residuals. All analyses were performed using R version 4.1.1 [16].

### Ethics

Ethical approval for the study was granted by NHS Greater Glasgow and Clyde Research and Development and the UK National Research Ethics Service (Reference 20/NS/0066). All participants provided written informed consent prior to enrolment.

## Results

One thousand three hundred and six patients were prospectively identified as being potentially eligible to participate in a longitudinal cohort study involving at least one visit post-discharge. Participants were recruited across three centres in the West of Scotland between 22 May 2020 and 16 March 2021. Of these individuals, 267 COVID-19 provided written informed consent to participate and 260 had longer term follow up data available. The median follow-up period was 1028 days (interquartile range (IQR) 1000, 1085). The original flow diagram of recruitment is provided as Supplementary Figure S1. There were 7 participants lost to follow-up.

### Baseline characteristics: cases and controls

All (*n* = 260) of the participants with COVID-19 had a positive polymerase chain reaction test and all the control group (*n* = 48) tested negative for COVID-19 serology. Baseline demographics of the control and COVID-19 cohort are detailed in Table 1. Standard care blood results are presented in Supplementary Table S1.

**Table 1:** Enrolment characteristics

		Control	COVID-19	p-value		COVID-19: Death or Unplanned Hospitalisation		p-value
						Yes	No	
**N**		**48**	**260**			**112**	**148**	
*Baseline Demographics*								
Age, years	Mean±SD	56.4±9.0	54.9±12.1	p = 0.424		55.1±13.9	54.8±10.6	p = 0.826
Female Sex	N (%)	28 (58.3%)	107 (41.2%)	p = 0.039		50 (44.6%)	57 (38.5%)	p = 0.373
Healthcare Worker	N (%)	18 (39.1%)	53 (20.4%)	p = 0.008		17 (15.2%)	36 (24.3%)	p = 0.087
Ethnicity	N (%) White N (%) Asian N (%) Other	41 (85.4%) 7 (14.6%) 0 (0.0%)	233 (89.6%) 21 (8.1%) 6 (2.3%)	p = 0.228		100 (89.3%) 9 (8.0%) 3 (2.7%)	133 (89.9%) 12 (8.1%) 3 (2.0%)	p = 1.000
Scottish Index Multiple Deprivation Quintile	N (%) Q1 - Most Deprived N (%) Q2 N (%) Q3 N (%) Q4 N (%) Q5 - Least Deprived	7 (14.6%) 7 (14.6%) 10 (20.8%) 14 (29.2%) 10 (20.8%)	102 (39.2%) 58 (22.3%) 31 (11.9%) 26 (10.0%) 43 (16.5%)	p < 0.001		45 (40.2%) 28 (25.0%) 14 (12.5%) 14 (12.5%) 11 (9.8%)	57 (38.5%) 30 (20.3%) 17 (11.5%) 12 (8.1%) 32 (21.6%)	p = 0.111
*Presenting Characteristics*								
Body mass index, kg/m^2^	Mean±SD	29.1±5.3	31.1±7.0	p = 0.061		31.4±7.4	30.9±6.6	p = 0.498
Heart Rate, bpm	Mean±SD	69±11	95±19	p < 0.001		96±16	94±21	p = 0.443
Systolic BP, mmHg	Mean±SD	140±17	130±19	p < 0.001		129±18	131±20	p = 0.305
Diastolic BP, mmHg	Mean±SD	81±12	77±13	p = 0.107		77±13	77±13	p = 0.963
Peripheral O_2_ saturation, %	Mean±SD	98±1	94±6	p < 0.001		94±5	93±6	p = 0.167
WHO Clinical severity score	N (%) Hospitalised, no O_2_ N (%)O_2_ (mask/nasal prongs) N (%) Non-inv. ventilation N (%) Mech. ventilation		76 (29.3%) 124 (47.9%) 35 (13.5%) 24 (9.3%)			34 (30.6%) 58 (52.3%) 11 (9.9%) 8 (7.2%)	42 (28.4%) 66 (44.6%) 24 (16.2%) 16 (10.8%)	p = 0.316
*COVID-19 diagnosis*								
PCR test	N (%)	0 (0.0%)	260 (100.0%)	-		112 (100.0%)	148 (100.0%)	-
Nosocomial	N (%)	0 (0.0%)	12 (4.6%)	-		6 (5.4%)	6 (4.1%)	p = 0.767
Chest x-ray/CT scan	N (%) Typical of COVID-19 N (%) Atypical of COVID-19 N (%) Unlikely N (%) Normal		181 (73.6%) 19 (7.7%) 7 (2.8%) 39 (15.9%)			71 (70.3%) 11 (10.9%) 3 (3.0%) 16 (15.8%)	110 (75.9%) 8 (5.5%) 4 (2.8%) 23 (15.9%)	p = 0.478
*Acute COVID-19 therapy*								
Oxygen	N (%)		183 (70.4%)			77 (68.8%)	106 (71.6%)	p = 0.681
Non-invasive respiratory support	N (%)		49 (18.8%)			12 (10.7%)	37 (25.0%)	p = 0.004
Invasive ventilation	N (%)		23 (8.8%)			8 (7.1%)	15 (10.1%)	p = 0.510
IV inotrope	N (%)		11 (4.2%)			4 (3.6%)	7 (4.7%)	p = 0.762
Antiviral	N (%)		66 (25.4%)			25 (22.3%)	41 (27.7%)	p = 0.388
Steroid	N (%)		132 (50.8%)			56 (50.0%)	76 (51.4%)	p = 0.900
ICU	N (%)		33 (12.7%)			12 (10.7%)	21 (14.2%)	p = 0.456
*Cardiovascular History*								
Smoking	N (%) Never N (%) Former N (%) Current	31 (64.6%) 14 (29.2%) 3 (6.2%)	166 (63.8%) 80 (30.8%) 14 (5.4%)	p = 0.929		60 (53.6%) 43 (38.4%) 9 (8.0%)	106 (71.6%) 37 (25.0%) 5 (3.4%)	p = 0.009
Hypercholesterolemia	N (%)	22 (45.8%)	121 (46.5%)	p = 1.000		55 (49.1%)	66 (44.6%)	p = 0.531
Hypertension	N (%)	18 (37.5%)	85 (32.7%)	p = 0.510		41 (36.6%)	44 (29.7%)	p = 0.286
Diabetes mellitus	N (%)	5 (10.4%)	59 (22.7%)	p = 0.055		30 (26.8%)	29 (19.6%)	p = 0.181
Chronic kidney disease	N (%)	0 (0.0%)	18 (6.9%)	p = 0.087		11 (9.8%)	7 (4.7%)	p = 0.140
Canadian Cardiovascular Society Angina Class	N (%) No Angina N (%) Angina Class I-IV	46 (100.0%) 0 (0.0%)	248 (95.4%) 12 (4.6%)	p = 0.225		106 (94.6%) 6 (5.4%)	142 (95.9%) 6 (4.1%)	p = 0.767
Heart failure	N (%)	0 (0.0%)	7 (2.7%)	p = 0.601		3 (2.7%)	4 (2.7%)	p = 1.000
Myocardial infarction	N (%)	0 (0.0%)	23 (8.8%)	p = 0.032		12 (10.7%)	11 (7.4%)	p = 0.384
Stroke or transient ischemic attack	N (%)	2 (4.2%)	7 (2.7%)	p = 0.635		4 (3.6%)	3 (2.0%)	p = 0.468
Peripheral vascular disease	N (%)	0 (0.0%)	3 (1.2%)	p = 1.000		1 (0.9%)	2 (1.4%)	p = 1.000
Previous PCI	N (%)	0 (0.0%)	10 (3.8%)	p = 0.371		5 (4.5%)	5 (3.4%)	p = 0.749
Previous CABG	N (%)	0 (0.0%)	4 (1.5%)	p = 1.000		0 (0.0%)	4 (2.7%)	p = 0.137
Cardiovascular disease/treatment	N (%)	21 (43.8%)	124 (47.7%)	p = 0.640		58 (51.8%)	66 (44.6%)	p = 0.260
*Risk Scores*								
ISARIC-4c (in-hosp mortality), %	Median (IQR)	2.3 (0.3, 7.8)	7.8 (4.8, 19.2)	p < 0.0001		7.8 (4.8, 19.2)	9.8 (4.8, 19.2)	p = 0.848
Q-Risk 3 (10y CV risk), %	Median (IQR)	7.0 (5.2, 15.7)	11.8 (5.0, 20.5)	p = 0.166		13.1 (6.4, 23.1)	11.4 (3.7, 18.0)	p = 0.074
Charlson Comorbidity Index	Median (IQR)	1 (0, 2)	2 (1, 3)	p = 0.120		2 (1, 3)	1 (0, 2)	p = 0.016
*Pre-existing maintenance medication*								
Aspirin	N (%)	1 (2.1%)	20 (7.7%)	p = 0.218		9 (8.0%)	11 (7.4%)	p = 1.000
Statin	N (%)	14 (29.2%)	70 (26.9%)	p = 0.728		31 (27.7%)	39 (26.4%)	p = 0.888
Beta-blocker	N (%)	2 (4.2%)	35 (13.5%)	p = 0.089		16 (14.3%)	19 (12.8%)	p = 0.855
ACE inhibitor	N (%)	6 (12.5%)	55 (21.2%)	p = 0.236		30 (26.8%)	25 (16.9%)	p = 0.066
ARB	N (%)	3 (6.2%)	19 (7.3%)	p = 1.000		10 (8.9%)	9 (6.1%)	p = 0.472
Oral anticoagulation	N (%)	1 (2.1%)	14 (5.4%)	p = 0.481		11 (9.8%)	3 (2.0%)	p = 0.010

Summaries shown for controls and COVID-19 patients. COVID-19 patients also split by incidence of death or unplanned hospitalisation (any cause) during long-term follow-up. Summaries are Mean ±sd, Median (IQR), or n (%). P-values from T-Test, kruskal-wallis test, or Fisher’s exact test

Compared to the control group, the post-COVID-19 group had similar mean±standard deviation (SD) age (54.9±12.1 vs. 56.4 ± 9.0 years; *p* = 0.424), body mass index (31.0±7.0 vs. 29.1 ± 5.3 kg/m^2^; *p* = 0.061), and ethnicity distribution (non-White ethnicity: 27 (10.4%) vs. 7 (14.6%); *p* = 0.228) but fewer were female (107 (41.2%) vs. 28 (58.3%); *p* = 0.039) or healthcare workers (53 (20.4%) vs. 18 (39.1%); *p* = 0.008).

Considering clinical characteristics, median (interquartile range) Charlson Comorbidity Index (2 (1, 3) vs. 1 (0, 2); *p* = 0.120), 10-year cardiovascular risk (Q-Risk-3 score) (11.8 (5.0,20.5) vs. 7.0 (5.2, 15.7); *p* = 0.166) and there were no statistically significant difference in smoking status between the control and COVID-19 participants (*p* = 0.929). *p* = 0.929). C-reactive protein was markedly higher in the COVID-19 group (119 (40, 190) vs. 2 (1, 4); *p* < 0.001) whereas haemoglobin level was similar (142 (133, 152) vs 142 (135, 151) g/L; *p* = 0.737).

### COVID-19 illness severity score

Considering the World Health Organization (WHO) Clinical Severity Score, 76 (29.3%) individuals were hospitalised without the need for oxygen therapy, 124 (47.9%) received oxygen therapy by mask or nasal prongs, 35 (13.5%) received non-invasive ventilation, and 24 (9.3%) received mechanical ventilation.

The ISARIC 4c percentage estimate of in-hospital mortality was 7.8 (4.8, 19.2) in the COVID-19 group and 2.3 (0.3, 7.8) in the control group (*p* < 0.001).

### Clinical outcomes after hospitalisation for COVID-19

**Fig. 1:**
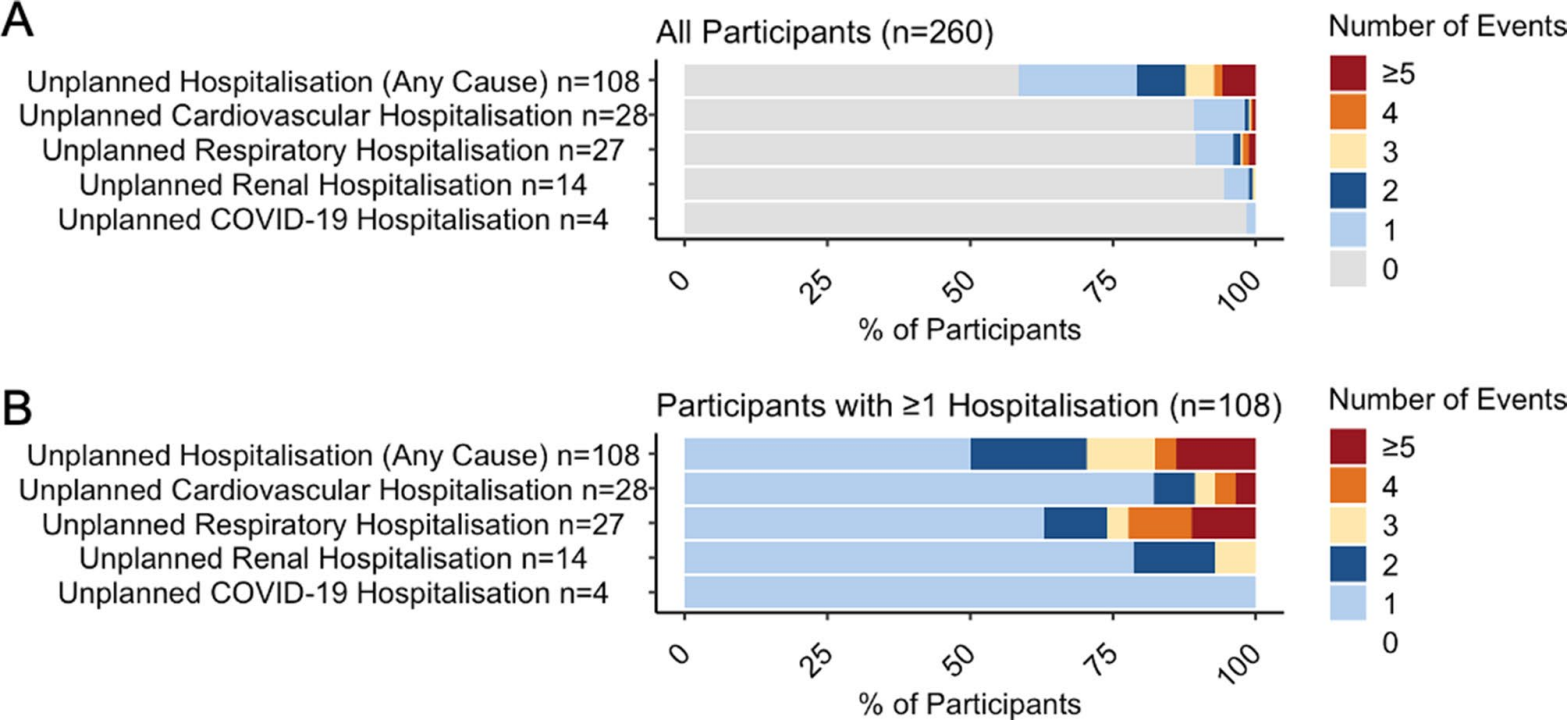
Unplanned hospitalisation stratified by event type. N on y-axis indicates the number of participants with ≥1 event. Colours represent the cumulative number of events per category. **A**. Proportion of participants experiencing each event type. **B**. Proportion of participants with ≥1 event, stratified by event type within each category

Clinical event rates are summarised in Table 2 for first events. Total events are reported in Supplementary Table S2. During the follow-up period, there were 6 (2.3%) deaths over 728.9 person-years of observation, corresponding to an incidence rate of 0.8 events per 100 person-years (95% confidence interval [CI]: 0.4, 1.8). At least one unplanned hospitalisation occurred in 108 (41.5%) participants over 591.6 person-years, resulting in an incidence rate of 18.3 events per 100 person-years (95% CI: 15.1, 22.0). When considering the composite outcome of all-cause mortality or unplanned hospitalisation, 112 first events were observed over 591.6 person-years, yielding an incidence rate of 18.9 events per 100 person-years (95% CI: 15.7, 22.8). When stratifying hospitalisation by type, 28 participants (10.8%) had at least one cardiovascular hospitalisation, 27 (10.4%) had at least one respiratory hospitalisation, and 14 (5.4%) had at least one renal hospitalisation. Among the 108 participants with at least 1 rehospitalisation, the first unplanned rehospitalisation was cardiovascular in 18 cases (16.7%), respiratory in 16 (14.8%), and renal in 4 (3.7%). One participant had a first rehospitalisation involving both cardiovascular and respiratory causes. An overall summary of rehospitalisations is shown in Fig. 1. Hospitalisations by all event type are detailed in Supplementary Table S3.

**Table 2:** Clinical outcomes including death and unplanned episodes of care in post-hospitalised COVID-19 population

	Number (%) of first events	Person-years of follow-up	First event rate (per 100py, with 95% CI)
*Deaths and unplanned hospitalisations*			
Death (Any Cause)	6 (2.3%)	728.9	0.8 (0.4, 1.8)
Unplanned Hospitalisation (Any Cause)	108 (41.5%)	591.6	18.3 (15.1, 22.0)
Death or (Unplanned) Hospitalisation (Any Cause)	112 (43.1%)	591.6	18.9 (15.7, 22.8)
Cardiovascular Death	1 (0.4%)	728.9	0.1 (0.0, 1.0)
Renal Death	0 (0.0%)	728.9	-
Respiratory Death	3 (1.2%)	728.9	0.4 (0.1, 1.3)
COVID-19 Death	1 (0.4%)	728.9	0.1 (0.0, 1.0)
Unplanned Cardiovascular Hospitalisation	28 (10.8%)	694.7	4.0 (2.8, 5.8)
Unplanned Renal Hospitalisation	14 (5.4%)	717.9	2.0 (1.2, 3.3)
Unplanned Respiratory Hospitalisation	27 (10.4%)	702.1	3.8 (2.6, 5.6)
Unplanned COVID-19 Hospitalisation	4 (1.5%)	724.9	0.6 (0.2, 1.5)
*Cardiovascular events*			
Myocardial infarction	7 (2.7%)	723.2	1.0 (0.5, 2.0)
Percutaneous Coronary Intervention	3 (1.2%)	723.7	0.4 (0.1, 1.3)
Coronary Artery Bypass Grafting	0 (0.0%)	728.9	-
Cerebrovascular accident	2 (0.8%)	724.8	0.3 (0.1, 1.1)
Heart Failure	5 (1.9%)	724.4	0.7 (0.3, 1.7)
Deep vein thrombosis	3 (1.2%)	728.3	0.4 (0.1, 1.3)
New atrial fibrillation	2 (0.8%)	725.3	0.3 (0.1, 1.1)
Ventricular tachycardia or fibrillation	0 (0.0%)	728.9	-
*Respiratory Outcomes*			
Pulmonary fibrosis	2 (0.8%)	728.7	0.3 (0.1, 1.1)
New diagnosis asthma	1 (0.4%)	728.9	0.1 (0.0, 1.0)
Pulmonary embolism	3 (1.2%)	723.0	0.4 (0.1, 1.3)
Long-term oxygen therapy	2 (0.8%)	727.2	0.3 (0.1, 1.1)

Person-years of follow-up calculated from date of index COVID-19 hospitalisation discharge to earliest of date of first event, date of death, or date of clinical record review. Event rate confidence interval estimated using Poisson regression model

Myocardial infarction was the most common cardiovascular event, occurring in 2.7% of individuals, with a first event rate of 1.0 per 100 person-years (95% CI: 0.5, 2.0). Heart failure occurred in 1.9% (0.7 per 100 person-years, 95% CI: 0.3, 1.7), while cerebrovascular accidents, deep vein thrombosis, new atrial fibrillation, and pulmonary embolism each affected 0.8–1.2% of participants, with event rates between 0.3 and 0.4 per 100 person-years. No cases of coronary artery bypass grafting or ventricular arrhythmias were reported.

Respiratory outcomes included pulmonary fibrosis (0.8%, 0.3 per 100 person-years, 95% CI: 0.1, 1.1), pulmonary embolism (1.2%, 0.4 per 100 person-years, 95% CI: 0.1, 1.3), and long-term oxygen therapy (0.8%, 0.3 per 100 person-years, 95% CI: 0.1, 1.1). New diagnoses of asthma occurred in 0.4% of participants, with an event rate of 0.1 per 100 person-years (95% CI: 0.0, 1.0).

#### Predictors of death or unplanned hospitalisation

**Fig. 2:**
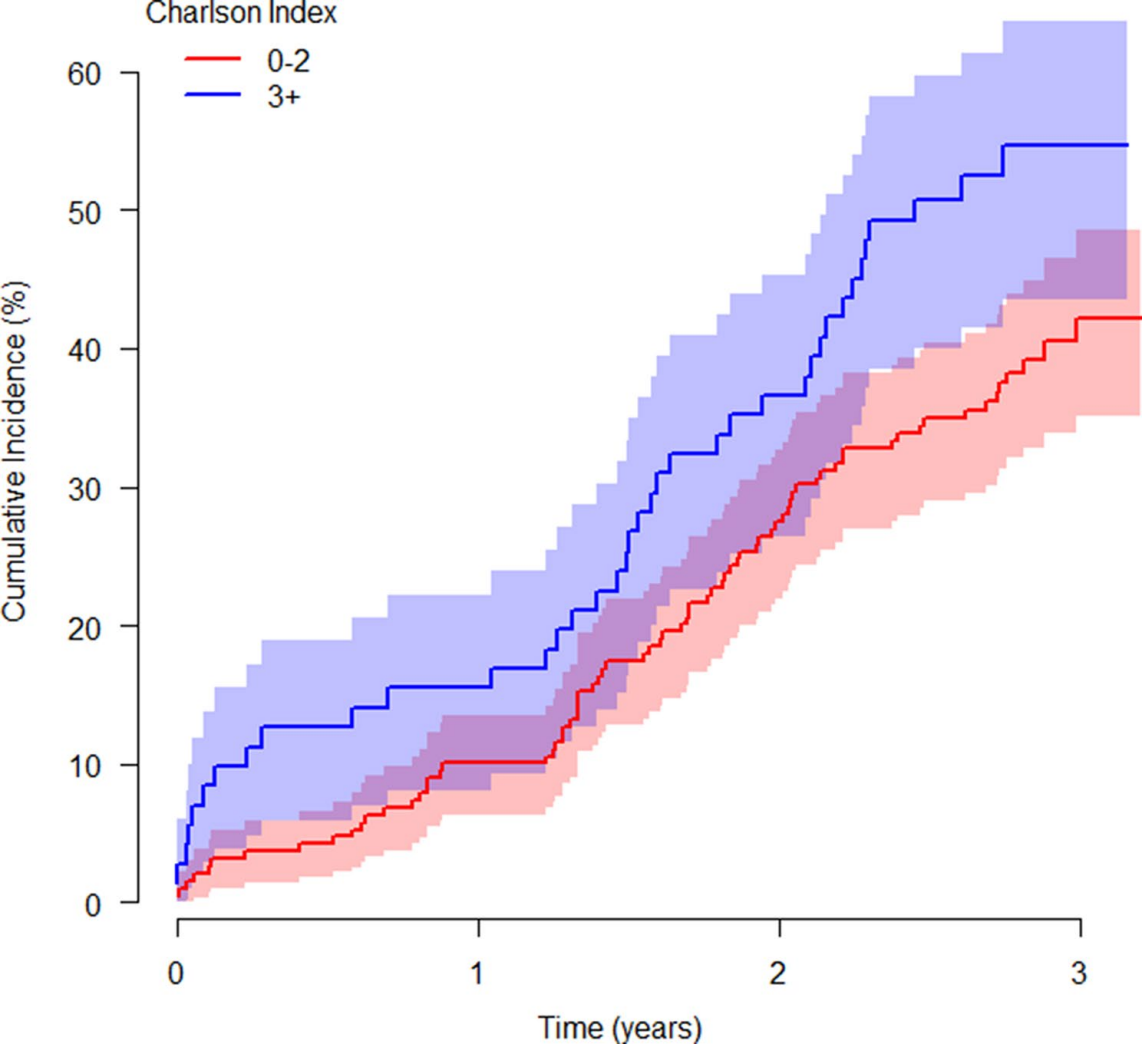
Kaplan-meir curve stratified by Charlson comorbidity index

Tests of the proportional hazards assumption in multivariable (*p* = 0.830) and best fit (*p* = 0.310) models gave no evidence of non-proportional hazards. The full model is presented in Supplementary Table S4. Multivariable predictors of death or unplanned hospitalisation (Table 3) were healthcare worker status (hazard ratio (95% confidence interval) 0.59 (0.34, 1.02); *p* = 0.046), Charlson Comorbidity Index (1.13 (1.02, 1.24); *p* = 0.020, Figure 2), cigarette smoking status (current vs. never 2.49 (1.21, 5.11) and former vs. never 1.31 (0.87, 1.98); *p* = 0.052) and haemoglobin (per 5 g/L) (0.93 (0.88, 0.99); *p* = 0.020).

**Table 3:** Multivariable, and best fitting predictors of risk of death or unplanned hospitalisation

Predictor		Univariate			Multivariable			Best Fit	
		HR (95% CI)	p-value		HR (95% CI)	p-value		HR (95% CI)	p-value
*Baseline Demographics*									
Healthcare Worker		0.59 (0.35, 0.99)	p = 0.034		0.69 (0.38, 1.26)	p = 0.211		0.59 (0.34, 1.02)	p = 0.046
*Acute COVID-19 therapy*									
Non-invasive respiratory support		0.43 (0.23, 0.78)	p = 0.002		0.69 (0.35, 1.33)	p = 0.248		0.40 (0.22, 0.74)	p = 0.001
*Cardiovascular History*									
Chronic kidney disease		1.89 (1.01, 3.52)	p = 0.066		1.05 (0.50, 2.20)	p = 0.901		-	
*Risk Scores*									
Q-Risk 3	per 5%	1.08 (1.00, 1.17)	p = 0.045		0.97 (0.86, 1.09)	p = 0.612		-	
Charlson Index	per point	1.17 (1.08, 1.27)	p = 0.001		1.13 (0.99, 1.29)	p = 0.086		1.13 (1.02, 1.24)	p = 0.020
*Pre-existing maintenance medication*									
ACE inhibitor		1.56 (1.02, 2.37)	p = 0.045		1.07 (0.64, 1.77)	p = 0.803		-	
OAC		3.40 (1.82, 6.35)	p = 0.001		1.90 (0.87, 4.17)	p = 0.126		-	
*Standard care blood results*									
*Full blood count*									
Haemoglobin	per 5 g/l	0.94 (0.88, 0.99)	p = 0.034		0.92 (0.86, 0.99)	p = 0.019		0.93 (0.88, 0.99)	p = 0.020
AST	per 10 IU/l	0.91 (0.85, 0.98)	p = 0.006		0.93 (0.80, 1.08)	p = 0.315		-	
ALT	per 10 IU/l	0.90 (0.83, 0.98)	p = 0.002		0.97 (0.85, 1.12)	p = 0.696		-	

Multivariable model includes all predictors with *p* < 0.10 in univariate analysis. Best fitting model derived by backward selection from multivariable model, at *p* < 0.10. Tests of the proportional hazards assumption in multivariable (*p* = 0.830) and best fit (*p* = 0.310) models gave no evidence of non-proportional hazards. P-values for categorical predictors with more than two levels represent global tests of association

By contrast, age (per 10-years: *p* = 0.599), female sex (*p* = 0.504), ethnicity (*p* = 0.917), SIMD (*p* = 0.096) and body mass index (per 5 kg/m^2^: *p* = 0.442) were not univariate predictors of death or unplanned hospitalisation (Supplementary Table S4). Considering the initial COVID-19 illness severity, WHO Clinical severity score (*p* = 0.187), ISARIC-4c percentage in-hospital mortality (*p* = 0.621), inflammation measured by initial C-reactive protein (*p* = 0.304) or estimated glomerular filtration rate (*p* = 0.794), were not predictors of death or unplanned hospitalisation. In addition, prior cardiovascular disease with binary classification also did not predict death or unplanned hospitalisation (*p* = 0.116).

## Discussion

At baseline, compared to controls, the participants who had been hospitalised for COVID-19 had similar age, body mass index, comorbidity and smoking history, but fewer women. During follow-up over 3-years, more than 4 in 10 cases were hospitalised or died.

Rehospitalisation was a frequent occurrence among patients following discharge from an initial admission with COVID-19, highlighting the ongoing burden on both individuals and healthcare services. Most of the hospitalisations were for a cardiovascular or respiratory reason.

Multivariable baseline predictors of death or unplanned hospitalisation included increasing Charlson Comorbidity Index, being a current smoker, or having a lower haemoglobin level (per 5 g/L category). This effect may be especially pertinent in the context of anaemia of chronic disease [17, 18], as comorbidity-based risk scores were also predictive of outcomes. Therefore, anaemia may partly capture the cumulative physiological impact of chronic illness which could be explored in larger COVID-19 cohort. In non-COVID-19 cohorts, anaemia of chronic disease has also been associated with higher risks of rehospitalisation and mortality, further supporting its role as both a marker of underlying morbidity and a potential contributor to poorer outcomes [19, 20]. Whether targeted management of anaemia of chronic disease would reduce the risk of rehospitalisation or death remains uncertain, as correcting the anaemia often requires addressing the underlying inflammatory or comorbid conditions rather than the haemoglobin level alone.

This association of healthcare worker status with rehospitalisations may reflect underlying differences in socioeconomic status, health literacy, or access to care among healthcare workers. These participants may engage with the healthcare system in qualitatively different ways. This also highlights that occupational exposure and the risk of persistent symptoms carry important implications for the safety and well-being of healthcare staff, as well as for workforce planning within the health service.

Interestingly, measures of the initial severity of COVID-19, including the WHO Clinical Severity Score, the ISARIC-4c percentage estimate of in-hospital mortality and C-reactive protein were not found to be predictive of death or hospitalisation. One possible explanation is that resource constraints and evolving clinical guidelines during the pandemic influenced treatment availability, making severity scores less reflective of long-term outcomes. Another reason for disease severity not being a univariate predictor is that the way severity is classified by the ISARIC-4c and WHO scores does not fully capture the complexity of a patient’s clinical trajectory. Instead, the specific pathway a patient followed through hospital such as whether they were admitted immediately to intensive care, received escalating care, or were discharged under particular conditions may be a more meaningful predictor that could be explored in other inpatient cohorts.

Earlier research has shown that variant clusters and transmission patterns vary by continent, implying that certain regions may have had a greater influence on the evolution of COVID-19 and global spread [21]. Consequently, findings in one country may not reflect those in other areas. Vaccination status may also influence hospitalisation rates. As we enter a new phase of the COVID-19 pandemic, it is sensible to examine rehospitalisation patterns in predominantly immunised populations.

The strengths of the CISCO-19 study cohort include prospective case selection and consent for electronic record linkage, allowing for near complete case follow up. In addition, the CISCO-19 screening criteria included a near all-comers approach.

These results highlight that in survivors of COVID-19, the initial health status as reflected by the Charlson Comorbidity Index, current smoking and haemoglobin level, are key predictors of longer-term outcomes, notably rehospitalisation as opposed to the clinical severity of COVID-19 at the time of hospitalisation. This is in keeping with predictors of rehospitalisation and mortality in general including comorbidity burden [22, 23] and may represent a contributing factor to rehospitalisation in the context of Long COVID [24, 25]. The results of this study emphasise the relevance of quantifying health status in hospitalised patients to inform risk stratification post-discharge and a need for targeted post-discharge care that considers both medical and socioeconomic risk factors including multimorbidity. Whether interventions that modify these characteristics might improve prognosis merits further research.

### Limitations

The cohort was predominantly unvaccinated at the time of their initial COVID-19 infection and study sites were limited to the West of Scotland. There is evidence to suggest vaccination strategies have varying effectiveness by variant type [26]. The longer-term impact of vaccination by variant type will be of interest to the clinical and public health community in the coming years.

This analysis was designed to focus on first events. As such, if participants experienced more than one hospitalisation in the same category, subsequent events would be precluded from inclusion in this analysis. The determinants of subsequent unplanned admissions are likely to differ meaningfully from those of the initial event, as later events may be influenced by changes in clinical status or treatment pathways following the first admission. Nonetheless, descriptive plots to illustrate these occurrences are provided in Fig. 1.

## Conclusion

This prospective, cohort study provides new insights into illness trajectory over 3-years following hospitalisation for COVID-19. Surprisingly, the severity of COVID-19 did not predict rehospitalisation or mortality. By contrast, co-morbidity, smoking status and haemoglobin were multivariable predictors of death or rehospitalisation. Some of these characteristics may be modifiable. Whether targeted interventions to modify these characteristics might improve prognosis merits further assessment.

## Electronic supplementary material

Below is the link to the electronic supplementary material.

Supplementary Material 1

## Data Availability

Data requests will be considered by the Steering Group, which includes representatives of the sponsor, the University of Glasgow, senior investigators independent of the research team and the chief investigator. The Steering Group will take account of the scientific rationale, ethics, logistics and resource implications. Data access requests should be initially submitted by email to the chief investigator (C.B., corresponding author). The source data include the de-identified numerical data used for the statistical analyses and de-identified imaging scans (MRI and CT) and ECGs. Data access will be provided through the secure analytical platform of the Robertson Centre for Biostatistics. This secure platform enables access to de-identified data for analytical purposes, without the possibility of removing the data from the server. Requests for transfer of de-identified data (including source imaging scans) will be considered by the Steering Group, and, if approved, a collaboration agreement would be expected. The Steering Group will consider any cost implications, and cost recovery would be expected on a not-for-profit basis.

## Abbreviations

ACE inhibitor: Angiotensin converting enzyme inhibitor
ARB: Angiotensin receptor blocker
CABG: Coronary artery bypass graft
CRP: C-reactive protein
HR: Hazard ratio
IQR: Interquartile range
ISARIC: International Severe Acute Respiratory and emerging Infection Consortium
PCI: Percutaneous intervention
SD: Standard deviation
WHO: World Health Organisation
