# Supplementary material for: Illness trajectory in the longer term after hospitalisation for COVID-19: a prospective, multicentre cohort study
Source: BMC Infect Dis. 2026 Jan 20;26:344. doi: 10.1186/s12879-025-12487-w (PMC12903569; doi:10.1186/s12879-025-12487-w)
Supplement: Supplementary file 1 — Supplementary Material 1 [file 12879_2025_12487_MOESM1_ESM.docx]

# Supplementary Material

**Figure S1. Flow diagram of recruitment during the CISCO-19 study to longer term follow up.**

#
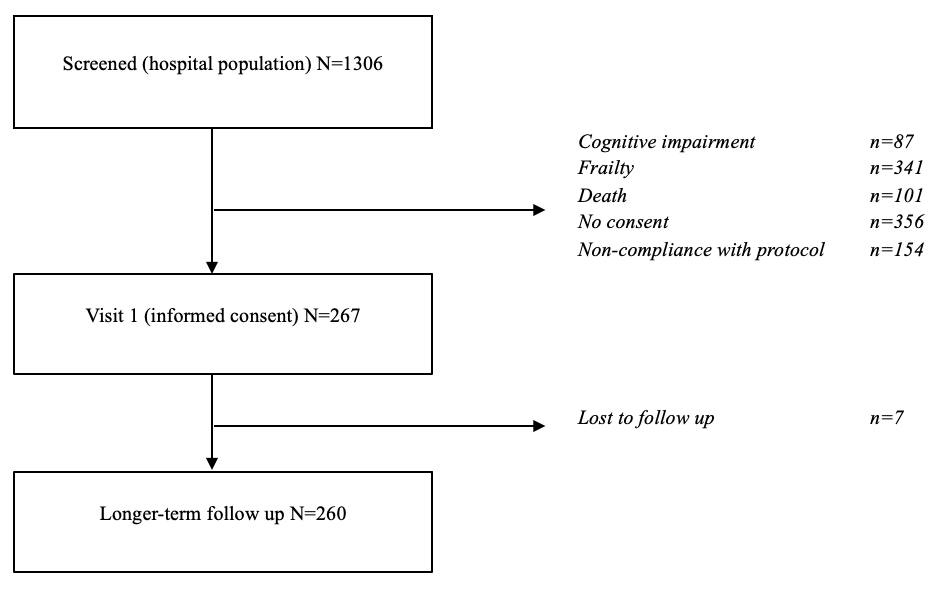


| **Table S1: Enrolment characteristics.** Summaries shown for controls and COVID-19 patients. COVID-19 patients also split by incidence of death or unplanned hospitalisation (any cause) during long-term follow-up. Summaries are Mean ±SD, Median (IQR), or N (%). P-values from T-Test, Kruskal-Wallis Test, or Fisher's Exact Test. | | | | | | | | |
| --- | --- | --- | --- | --- | --- | --- | --- | --- |
|  |  | Control | COVID-19 | p-value |  | COVID-19: Death or Unplanned Hospitalisation | | p-value |
|  |  |  |  |  |  | Yes | No |  |
| N |  | 48 | 260 |  |  | 112 | 148 |  |
| *Standard care blood results* | | | | | | | | |
| *Full blood count* | | | | | | | | |
| Haemoglobin, g/l | Median (IQR) | 142 (135, 151) | 142 (133, 152) | p=0.737 |  | 140 (131, 149) | 144 (134, 153) | p=0.058 |
| White cell count, 10^9^/l | Median (IQR) | 6.90 (5.85, 7.70) | 6.50 (4.97, 8.40) | p=0.398 |  | 6.35 (5.10, 8.45) | 6.75 (4.80, 8.40) | p=0.786 |
| Platelet count, 10^9^/l | Median (IQR) | 257 (216, 316) | 216 (181, 272) | p<0.001 |  | 216 (180, 272) | 216 (184, 271) | p=0.701 |
| Neutrophil count, 10^9^/l | Median (IQR) | 3.75 (3.18, 4.65) | 4.65 (3.50, 6.50) | p=0.003 |  | 4.70 (3.50, 6.50) | 4.60 (3.40, 6.43) | p=0.553 |
| Lymphocyte count, 10^9^/l | Median (IQR) | 2.10 (1.70, 2.40) | 1.00 (0.78, 1.42) | p<0.001 |  | 1.00 (0.80, 1.40) | 1.00 (0.70, 1.50) | p=0.711 |
| Neutrophil/Lymphocyte ratio | Median (IQR) | 1.79 (1.60, 2.27) | 4.74 (2.74, 7.51) | p<0.001 |  | 5.19 (2.91, 7.57) | 4.37 (2.66, 7.40) | p=0.344 |
| Hematocrit | Mean±SD | 0.43±0.03 | 0.42±0.04 | p=0.208 |  | 0.42±0.05 | 0.42±0.04 | p=0.242 |
| *U&E* | | | | | | | | |
| Sodium, mmol/l | Mean±SD | 139.2±2.6 | 136.4±3.7 | p<0.001 |  | 136.5±3.8 | 136.4±3.7 | p=0.723 |
| Potassium, mmol/l | Mean±SD | 4.09±0.31 | 4.02±0.45 | p=0.301 |  | 4.00±0.49 | 4.03±0.41 | p=0.660 |
| Urea, mmol/l | Mean±SD | 5.51±1.53 | 6.30±6.72 | p=0.418 |  | 5.92±3.27 | 6.59±8.45 | p=0.432 |
| Creatinine, mmol/l | Median (IQR) | 70 (62, 78) | 76 (62, 94) | p=0.0520 |  | 76 (62, 91) | 77 (61, 95) | p=0.624 |
| eGFR, ml/min/1.73m^2^ | Median (IQR) | 92 (77, 102) | 92 (72, 103) | p=0.538 |  | 91 (70, 103) | 92 (72, 102) | p=0.871 |
| eGFR <60 ml/min/1.73m^2^ | N (%) | 1 (2.1%) | 35 (13.5%) | p=0.025 |  | 16 (14.3%) | 19 (12.8%) | p=0.855 |
| Glucose, μmol/l | Median (IQR) | 5.0 (4.4, 6.4) | 6.4 (5.6, 7.4) | p<0.001 |  | 6.1 (5.4, 7.3) | 6.5 (5.7, 7.4) | p=0.269 |
| *LFTs* | | | | | | | | |
| Bilirubin, μmol/l | Median (IQR) | 9.0 (6.8, 11.2) | 9.0 (7.0, 13.0) | p=0.528 |  | 9.0 (6.0, 12.0) | 9.5 (7.0, 14.0) | p=0.183 |
| AST, IU/l | Median (IQR) | 23.0 (20.0, 26.8) | 34.0 (23.0, 52.2) | p<0.001 |  | 32.0 (22.8, 44.2) | 37.0 (24.0, 58.0) | p=0.046 |
| ALT, IU/l | Median (IQR) | 22.5 (17.8, 32.0) | 30.5 (21.0, 44.2) | p=0.002 |  | 27.0 (20.0, 38.2) | 32.5 (23.0, 51.5) | p=0.011 |
| Alk Phos, IU/l | Median (IQR) | 79.0 (71.0, 94.2) | 78.0 (63.0, 99.0) | p=0.688 |  | 78.0 (65.8, 98.2) | 79.0 (61.8, 99.2) | p=0.818 |
| Albumin, g/l | Median (IQR) | 41.5 (40.0, 43.2) | 34.0 (31.0, 38.0) | p<0.001 |  | 34.0 (31.0, 38.0) | 34.0 (31.0, 37.2) | p=0.670 |
| *C-rP* | | | | | | | | |
| CRP, mg/l | Median (IQR) | 2 (1, 4) | 119 (40, 190) | p<0.001 |  | 99 (32, 188) | 130 (50, 194) | p=0.163 |

| **Table S2: Clinical outcomes including death and unplanned episodes of care in post-hospitalised COVID19 population. Person-years of follow-up calculated from date of index COVID-19 hospitalisation discharge to earliest of date of death, or date of clinical record review. Event rate confidence interval estimated using negative binomial regression model.** | | | |
| --- | --- | --- | --- |
|  | Total (Mean) Number of events | Total person-years of follow-up | Total event rate (per 100py, with 95% CI) |
| *Deaths and unplanned hospitalisations* | | | |
| Unplanned Hospitalisation (Any Cause) | 252 (0.97) | 728.9 | 34.9 (28.2, 43.4) |
| Death or (Unplanned) Hospitalisation (Any Cause) | 258 (0.99) | 728.9 | 36.8 (29.7, 45.6) |
| Unplanned Cardiovascular Hospitalisation | 39 (0.15) | 728.9 | 5.4 (3.6, 8.1) |
| Unplanned Renal Hospitalisation | 18 (0.07) | 728.9 | 2.6 (1.4, 4.7) |
| Unplanned Respiratory Hospitalisation | 56 (0.22) | 728.9 | 7.6 (4.7, 12.4) |
| Unplanned COVID-19 Hospitalisation | 4 (0.02) | 728.9 | 0.5 (0.2, 1.5) |

| **Table S3. Adjudicated Classification of Unplanned Hospitalisations by Clinical Category** | | | | | |
| --- | --- | --- | --- | --- | --- |
|  | Total | Cardiovascular | Renal | Respiratory | COVID-19 |
| Total Hospitalisations | 252 | 39 | 18 | 56 | 4 |
| Cardiovascular | 39 | 39 | 0 | 2 | 0 |
| Respiratory | 56 | 2 | 1 | 56 | 3 |
| Renal | 18 | 0 | 18 | 1 | 0 |
| Gastroenterology | 22 | 0 | 2 | 0 | 0 |
| Neurology | 12 | 0 | 0 | 2 | 0 |
| General Surgery/Urology | 20 | 0 | 1 | 0 | 0 |
| Orthopedics | 21 | 1 | 0 | 3 | 0 |
| Traumatic injury | 62 | 0 | 0 | 2 | 0 |
| Hematology | 4 | 0 | 0 | 0 | 0 |
| Endocrinology | 8 | 1 | 2 | 1 | 0 |
| Ear, nose, throat | 6 | 0 | 0 | 0 | 0 |
| Ophthalmology | 1 | 0 | 0 | 0 | 0 |
| Rheumatology | 7 | 1 | 0 | 1 | 0 |
| Oncology | 1 | 0 | 0 | 0 | 0 |
| Psychiatry | 1 | 0 | 0 | 0 | 0 |
| Infectious diseases | 7 | 0 | 0 | 4 | 4 |
| Dermatology | 1 | 0 | 0 | 0 | 0 |

| **Table S4: Univariate, multivariable, and best fitting predictors of risk of death or unplanned hospitalisation.** Multivariable model includes all predictors with p<0.10 in univariate analysis. Best fitting model derived by backward selection from multivariable model, at p<0.10. Tests of the proportional hazards assumption in multivariable (p=0.830) and best fit (p=0.310) models gave no evidence of non-proportional hazards. P-values for categorical predictors with more than two levels represent global tests of association. | | | | | | | | | |
| --- | --- | --- | --- | --- | --- | --- | --- | --- | --- |
| Predictor |  | Univariate | |  | Multivariable | |  | Best Fit | |
|  |  | HR (95% CI) | p-value |  | HR (95% CI) | p-value |  | HR (95% CI) | p-value |
| *Baseline Demographics* | | | | | | | | | |
| Age | per 10 years | 1.04 (0.89, 1.23) | p=0.600 |  | - |  |  | - |  |
| Female Sex |  | 1.14 (0.78, 1.65) | p=0.504 |  | - |  |  | - |  |
| Healthcare Worker |  | 0.59 (0.35, 0.99) | p=0.034 |  | 0.69 (0.38, 1.26) | p=0.2113 |  | 0.59 (0.34, 1.02) | p=0.046 |
| Ethnicity | Asian vs. White Other vs. White | 1.11 (0.56, 2.20) 1.20 (0.38, 3.79) | p=0.917 |  | - |  |  | - |  |
| Scottish Index of Multiple Deprivation Quintile | Q2 vs. Q1 Q3 vs. Q1 Q4 vs. Q1 Q5 vs. Q1 | 1.09 (0.68, 1.74) 0.96 (0.53, 1.75) 1.42 (0.78, 2.58) 0.51 (0.26, 0.98) | p=0.096 |  | 1.11 (0.63, 1.94) 0.86 (0.42, 1.76) 1.26 (0.58, 2.74) 0.57 (0.27, 1.19) | p=0.3799 |  | 1.23 (0.75, 2.02) 0.88 (0.47, 1.63) 1.58 (0.84, 2.98) 0.57 (0.29, 1.11) | p=0.087 |
| *Presenting Characteristics* | | | | | | | | | |
| Weight | per 10kg | 1.00 (0.91, 1.10) | p=0.975 |  | - |  |  | - |  |
| Height | per 10cm | 0.92 (0.77, 1.09) | p=0.335 |  | - |  |  | - |  |
| Body mass index | per 5kg/m^2^ | 1.05 (0.92, 1.20) | p=0.442 |  | - |  |  | - |  |
| Body surface area | per 0.5m^2^ | 0.97 (0.67, 1.40) | p=0.862 |  | - |  |  | - |  |
| Heart Rate | per 10bpm | 1.03 (0.94, 1.13) | p=0.500 |  | - |  |  | - |  |
| Systolic BP | per 10mmHg | 0.96 (0.87, 1.05) | p=0.368 |  | - |  |  | - |  |
| Diastolic BP | per 10mmHg | 1.01 (0.88, 1.17) | p=0.857 |  | - |  |  | - |  |
| SPO_2_ | per % | 1.02 (0.99, 1.06) | p=0.166 |  | - |  |  | - |  |
| Respiratory rate | per 5/min | 0.95 (0.85, 1.06) | p=0.307 |  | - |  |  | - |  |
| WHO Clinical severity score | O_2_ vs. No O_2_ NI Vent vs. No O_2_ Mech Vent vs. No O_2_ | 1.13 (0.74, 1.72) 0.65 (0.33, 1.29) 0.64 (0.30, 1.39) | p=0.187 |  | - |  |  | - |  |
| *COVID-19 diagnosis* | | | | | | | | | |
| Nosocomial |  | 1.14 (0.50, 2.60) | p=0.756 |  | - |  |  | - |  |
| CXR/CT | Typical vs. Other | 0.81 (0.53, 1.25) | p=0.355 |  | - |  |  | - |  |
| *Acute COVID-19 therapy* | | | | | | | | | |
| Oxygen |  | 0.94 (0.63, 1.40) | p=0.768 |  | - |  |  | - |  |
| Non-invasive respiratory support |  | 0.43 (0.23, 0.78) | p=0.002 |  | 0.69 (0.35, 1.33) | p=0.248 |  | 0.40 (0.22, 0.74) | p=0.001 |
| Invasive ventilation |  | 0.67 (0.32, 1.37) | p=0.241 |  | - |  |  | - |  |
| IV inotrope |  | 0.67 (0.25, 1.83) | p=0.410 |  | - |  |  | - |  |
| Antiviral |  | 0.84 (0.54, 1.31) | p=0.426 |  | - |  |  | - |  |
| Steroid |  | 1.07 (0.73, 1.55) | p=0.732 |  | - |  |  | - |  |
| ICU |  | 0.71 (0.39, 1.29) | p=0.236 |  | - |  |  | - |  |
| *Cardiovascular History* | | | | | | | | | |
| Smoking | Former vs. Never Current vs. Never | 1.67 (1.13, 2.47) 2.18 (1.08, 4.38) | p=0.012 |  | 1.19 (0.74, 1.93) 2.43 (1.16, 5.09) | p=0.093 |  | 1.31 (0.87, 1.98) 2.49 (1.21, 5.11) | p=0.052 |
| Hypercholesterolemia |  | 1.25 (0.86, 1.81) | p=0.236 |  | - |  |  | - |  |
| Hypertension |  | 1.28 (0.87, 1.87) | p=0.220 |  | - |  |  | - |  |
| Diabetes |  | 1.43 (0.94, 2.18) | p=0.102 |  | - |  |  | - |  |
| Chronic kidney disease |  | 1.89 (1.01, 3.52) | p=0.066 |  | 1.05 (0.50, 2.20) | p=0.901 |  | - |  |
| Canadian cardiovascular society Angina Class |  | 1.19 (0.52, 2.71) | p=0.688 |  | - |  |  | - |  |
| Heart failure |  | 1.21 (0.38, 3.82) | p=0.751 |  | - |  |  | - |  |
| Myocardial infarction |  | 1.44 (0.79, 2.63) | p=0.254 |  | - |  |  | - |  |
| Stroke or transient ischemic attack |  | 1.58 (0.58, 4.28) | p=0.404 |  | - |  |  | - |  |
| Peripheral vascular disease |  | 0.74 (0.10, 5.33) | p=0.758 |  | - |  |  | - |  |
| Previous PCI |  | 1.25 (0.51, 3.06) | p=0.639 |  | - |  |  | - |  |
| Cardiovascular disease/treatment |  | 1.35 (0.93, 1.95) | p=0.116 |  | - |  |  | - |  |
| *Risk Scores* | | | | | | | | | |
| ISARIC-4c | per 5% | 1.02 (0.94, 1.11) | p=0.621 |  | - |  |  | - |  |
| Q-Risk 3 | per 5% | 1.08 (1.00, 1.17) | p=0.045 |  | 0.97 (0.86, 1.09) | p=0.612 |  | - |  |
| Charlson Index | per point | 1.17 (1.08, 1.27) | p=0.001 |  | 1.13 (0.99, 1.29) | p=0.086 |  | 1.13 (1.02, 1.24) | p=0.020 |
| *Pre-existing maintenance medication* | | | | | | | | | |
| Aspirin |  | 1.18 (0.60, 2.33) | p=0.645 |  | - |  |  | - |  |
| Statin |  | 1.15 (0.76, 1.73) | p=0.523 |  | - |  |  | - |  |
| Beta-blocker |  | 1.09 (0.64, 1.85) | p=0.754 |  | - |  |  | - |  |
| ACE inhibitor |  | 1.56 (1.02, 2.37) | p=0.045 |  | 1.07 (0.64, 1.77) | p=0.803 |  | - |  |
| ARB |  | 1.50 (0.78, 2.87) | p=0.247 |  | - |  |  | - |  |
| OAC |  | 3.40 (1.82, 6.35) | p=0.001 |  | 1.90 (0.87, 4.17) | p=0.126 |  | - |  |
| *Standard care blood results* | | | | | | | | | |
| *Full blood count* | | | | | | | | | |
| Haemoglobin | per 5g/l | 0.94 (0.88, 0.99) | p=0.034 |  | 0.92 (0.86, 0.99) | p=0.019 |  | 0.93 (0.88, 0.99) | p=0.020 |
| White cell count | per 10^9^/l | 1.00 (0.96, 1.04) | p=0.845 |  | - |  |  | - |  |
| Platelet count | per 10x10^9^/l | 0.99 (0.97, 1.01) | p=0.336 |  | - |  |  | - |  |
| Neutrophil count | per 10^9^/l | 1.03 (0.97, 1.09) | p=0.375 |  | - |  |  | - |  |
| Lymphocyte count | per 0.5x10^9^/l | 0.95 (0.82, 1.10) | p=0.202 |  | - |  |  | - |  |
| Neutrophil/Lymphocyte ratio | per unit | 1.02 (0.98, 1.05) | p=0.352 |  | - |  |  | - |  |
| Hematocrit | per 0.1 increase | 0.78 (0.51, 1.19) | p=0.260 |  | - |  |  | - |  |
| *U&E* | | | | | | | | | |
| Sodium | per mmol/l | 1.01 (0.96, 1.06) | p=0.765 |  | - |  |  | - |  |
| Potassium | per 0.1mmol/l | 0.99 (0.95, 1.04) | p=0.765 |  | - |  |  | - |  |
| Urea | per mmol/l | 0.99 (0.95, 1.03) | p=0.546 |  | - |  |  | - |  |
| Creatinine | per 10mmol/l | 0.99 (0.94, 1.05) | p=0.759 |  | - |  |  | - |  |
| eGFR | per 10ml/min/1.73m^2^ | 0.99 (0.91, 1.07) | p=0.794 |  | - |  |  | - |  |
| eGFR <60 ml/min/1.73m^2^ |  | 1.28 (0.75, 2.17) | p=0.375 |  | - |  |  | - |  |
| Glucose | per 5μmol/l | 1.09 (0.88, 1.35) | p=0.469 |  | - |  |  | - |  |
| *LFTs* | | | | | | | | | |
| Bilirubin | per 5μmol/l | 0.93 (0.79, 1.10) | p=0.397 |  | - |  |  | - |  |
| AST | per 10 IU/l | 0.91 (0.85, 0.98) | p=0.006 |  | 0.93 (0.80, 1.08) | p=0.315 |  | - |  |
| ALT | per 10 IU/l | 0.90 (0.83, 0.98) | p=0.002 |  | 0.97 (0.85, 1.12) | p=0.696 |  | - |  |
| Alk Phos | per 50 IU/l | 1.10 (0.99, 1.23) | p=0.115 |  | - |  |  | - |  |
| Albumin | per 5g/l | 1.01 (0.83, 1.22) | p=0.948 |  | - |  |  | - |  |
| *C-rP* | | | | | | | | | |
| CRP | per 50mg/l | 0.96 (0.88, 1.04) | p=0.305 |  | - |  |  | - |  |
